# 

**Published:** 1920-09

**Authors:** 


### Bristol Medico-Chirurgical Society.

---

**The Library is now open from 9 a.m. to 7 p.m.
except on Saturday, when it closes at 1 p.m.**

*September 21st,* 1920.

*The following donations have been received since the publication of the list in June.*

| | |
|---|---|
| D. S. Davies, M.D. (1) .. .. .. .. | 4 volumes. |
| J. M. Fortescue-Brickdale, M.D. (2) .. .. | 1 volume. |
| The Medical Research Committee (3) .. .. | 5 volumes. |
| C. Singer, M.D. (4) .. .. .. .. | 1 volume. |
| Lt.-Col. A. J. Sturmer (5) .. .. .. | 9 volumes. |
| J. O. Symes, M.D. (6) .. .. .. .. | 2 ,, |
| C. H. Walker, M.B. (7) .. .. .. .. | 1 volume. |
| John Wright & Sons Ltd. (8) .. .. .. | 16 volumes. |

### THE ONE HUNDRED AND FIRST LIST OF BOOKS.

The figures in brackets refer to the figures after the names of the donors, and show by whom the volumes were presented. The books to which no such figures are attached have either been bought from the Library Fund or received through the *Journal.*

| | | | |
|---|---|---|---|
| **Abernethy, J.** .. .. | *Local Diseases and Aneurisms* .. | 11th Ed. | 1829 |
| *Acute Intestinal Infections, Laboratory Diagnosis of* .. .. .. | | (3) | 1920 |
| **Alquier, P.** .. .. | *L'Appareillage dans les Fractures de Guerre* | (8) | 1917 |
| **Arcelin, F.** .. .. | *Exploration radiologique des Voies urinaires* | (8) | 1917 |
| **Armand-Delille, P.** | *Le Paludisme Macedonien* .. .. .. | (8) | 1917 |
| **Babinski, J.** .. .. | *Exposé des Travaux Scientifiques* .. .. .. | | 1913 |
| **Ballance, Sir C. A.** | *Surgery of the Heart* .. .. .. .. .. | | 1920 |
| **Barjon, F.** .. .. | *Radiodiagnostic des Affections pleuro-pulmonaires* .. .. .. .. .. | (8) | 1916 |
| **Berkeley, C.** .. .. | *Handbook of Midwifery* .. .. .. | 5th Ed. | 1920 |

**Bertin, P.** *Premières Heures du Blessé de Guerre* (8) 1918
**Blackham, R. J.** *Military Sanitation* 3rd Ed. [N.D.]
**Bourgeois, H.** *Otites et Surdités de Guerre* (8) 1917
**Burt, J. G. M.** *Illustrations of Surgical Anatomy* 2nd Ed. [1833]
**Buxton, D. W.** *Anæsthetics* 6th Ed. 1920
**Carmichael, R.** *Essay on Venereal Disease* 1814
**Chatelin, C.** *Les Blessures du Cerveau* (8) 2me Éd. 1918
*Chemical Warfare Medical Committee, Reports of the* (3) 1917–20
**Cooper, Sir A. P.** *Dislocations and Fractures* (Ed. by A. C. Lee) Abr. Ed. 1841
**Desfosses, P.** *La Suspension dans le Traitement des Fractures* (8) 1918
**Duval, P.** *Les Plaies de Guerre du Poumon* (8) 1917
**Fawdington, T.** *A Case of Melanosis* 1826
*Flying, The Medical Problems of* (3) 1920
**Gerste, A.** *Sur la Médecine et la Botanique des anciens Mexicians* 2me Éd. 1910
**Gilbert, A.** *Clinique médicale de l'Hôtel Dieu de Paris* (2) [1912]
**Gjessing, H. G. A.** *Kliniske Linsestudier* 1920
**Goodall-Copestake, B. M.** *Theory and Practice of Massage.* 3rd Ed. 1920
**Gregoire, R.** *Plaies de la Plevre et du Poumon* (8) 1917
**Gullan, M. A.** *Theory and Practice of Nursing* 1920
**Hutchison and H. Rainy, R.** *Clinical Methods* 7th Ed. 1920
**Ingleby, J. T.** *Facts and Cases in Obstetric Medicine* [N.D.]
**Ker, C. B.** *Infectious Diseases* 2nd Ed. 1920
**Kidd, F.** *Common Infections of the Kidneys* 1920
**Lagrange, F.** *Les Fractures de l'Orbite* (8) 1917
**Lumb, N.** *Gonococcal Infection in the Male* 1920
**Martel, T. de** *Traitement opératoire des Plaies du Crane* (8) 2me Éd. 1918
**Martindale and W. W. Westcott, W. H.** *The Extra Pharmacopœia.* Vol. I. 17th Ed. 1920
**Ombrédame, L.** *Localisation et Extraction des Projectiles* (8) 1918
**Policard, A.** *Evolution de la Plaie de Guerre* (8) 1918
**Ramsay, A. M.** *Clinical Ophthalmology* 1920
**Snell, J.** *Practical Guide to Operations on the Teeth* 1831
**Tidy, H. L.** *A Synopsis of Medicine* 1920
*Ventilation and Open-air Treatment, The Science of.* Pt. II. (3) 1920
**Willis, W. N.** *Wedded Love or Married Misery* [N.D.]
**Wolbach, S. B.** *Studies on Rocky Mountain Spotted Fever* 1919
**Woodwark, A. S.** *Manual of Medicine* 2nd Ed. 1920
**Zimmern, A.** *Electrodiagnostic de Guerre* (8) 1917

### TRANSACTIONS, REPORTS, JOURNALS, &c.

American Association of Genito-Urinary Surgeons, Transactions of the Vol. XII. 1919

American Association of Obstetricians and Gynecologists, Transactions of the .. .. .. .. .. .. .. .. Vol. XXXII. 1919
American Pediatric Society, Transactions of the Vol. XXXI. 1919
Archives of the Roentgen Ray .. .. .. .. .. .. Vol. XIX. 1914–15
Bristol Health Report for 1914 and 1916 .. .. .. .. .. (1) 1915–17
Bristol Port Sanitary District, Medical Officer's Report for 1914 and 1916 .. .. .. .. .. .. .. .. .. .. .. .. .. .. .. (1) 1915–17
Bristol Medico-Chirurgical Journal, The Vols. XXXIV.–XXXVI. 1916–19
British Journal of Ophthalmology, The .. .. .. (7) Vol. III. 1919
British Medical Journal, The .. .. .. .. .. .. Vol. I. for 1920
Department of Experimental Surgery, New York Hospital, Collected Reprints from .. .. .. .. .. .. .. .. .. Vol. II. 1915–19
English Catalogue of Books for 1916 and 1917 .. .. .. .. .. 1917–18
Indian Annals of Medical Science, The.. .. .. (4) Vol. XIX. 1877
Johns Hopkins Hospital Bulletin .. Vols. XXVIII., 1917; XXX., 1919
Journal of Experimental Medicine, The Vols. XXIX.–XXXI. 1919–20
Journal of Laryngology, Rhinology and Otology .. Vol. XXXIV. 1919
Journal of Medical Research, The .. .. Vols. XXXIX., XL. 1918–19
Journal of Obstetrics and Gynæcology of the British Empire (5) Vols. I.–IX. 1902–06
Lancet, The .. .. .. .. .. .. .. .. .. .. .. Vol. I. for 1920
Medical Clinics of North America .. .. (6) Vols. I., II., 1917–19
Medical Science: Abstracts and Reviews .. .. .. Vol. I. 1919–20
Royal Society of Medicine, Proceedings of the .. .. Vol. XII. 1919

---

### Obituary.

### Local Medical Notes.

THE INTERIM REPORT OF THE CONSULTATIVE COUNCIL UNDER THE MINISTRY OF HEALTH.

**Lord Dawson's Visit.**—On July 9th Lord Dawson, Chairman of the Consultative Council, was the chief guest at a luncheon at the Royal West of England Academy, when upwards of eighty representatives of the committees of hospitals and other medical institutions in the Counties of Bristol, Somerset, Wilts, Devonshire, Gloucester, etc., accepted the invitation of the President of the Bath and Bristol Branch of the British Medical Association and Mrs. Watson-Williams. Of those invited to meet Lord Dawson the following may be mentioned from :—

*Bristol.*—The Right Hon. the Lord Mayor, the Very Rev. the Dean of Bristol, the Presidents, the Chairmen of Committee, and Hon. Treasurers of the Royal Infirmary, General Hospital, Royal Hospital for Children, the Bristol Eye Hospital, the Homœopathic Hospital, the Master of the Society of Merchant Venturers, the Vice-Chancellor, Pro-Chancellor, and Pro-Vice-Chancellor of the University, the Chairman of the Education

Committee, the Hon. Sec. of the Bristol Branch of the Red Cross, Dr. Shingleton Smith, Mr. F. R. Cross, and many other representative medical practitioners.

*Cardiff.*—The Right Hon. the Lord Mayor and Dr. Maclean.

*Hereford.*—The Mayor and the late Mayor.

*Bath, Taunton, Exeter, Salisbury, Gloucester, Cheltenham.*—The Presidents and other representatives of all the hospitals, and the Medical Officers of Health of Bristol, Bath Rural, Gloucester, and Wilts, and many others.

Dr. Watson-Williams, in proposing the toast of "The Consultative Council," said that the presence of so many of the lay representatives of hospitals as well as medical colleagues to support him in welcoming Lord Dawson was indicative of the profound interest aroused on all hands in the Interim Report, and of the earnest desire of those who were responsible for organisations and hospitals for the relief of the sick, to take part in developing schemes for improving on the means which hitherto had served such a useful purpose. The Consultative Council was set up to advise and to assist the Ministry of Health, and in affording the medical profession and those whose work lay in the administration of Hospitals the opportunity of considering the Interim Report, they doubtless hoped for assistance in framing their final conclusions and report. It was interesting to note that it was the British Medical Association which initiated the movement culminating in the creation of a Ministry of Health, and it was in the West of England that this movement had its birth. Dr. Rumsey, of Cheltenham, by a paper read in 1867, led to the formation of a Committee, and as a result a deputation of the British Medical Association, including Dr. Symonds of Bristol and others from Bath and Cheltenham, waited on certain members of the Government in 1868 to ask for a Royal Commission on the chaotic state of Public Health Administration. On June 6th, 1868, the *British Medical Journal* urged the "appointment of a Minister of Health," and for fifty years the British Medical Association reiterated its plea, till at long length we now had the Ministry.

Lord Dawson, in replying, referred to the great movement in the early eighteenth century for the establishment of voluntary hospitals, Bristol being one of the first cities to rise to the occasion with the setting up of the Bristol Royal Infirmary. A new chapter was opening in the story of the hospitals of this country, and the West of England was once more in the forefront. Already in the County of Gloucester a scheme had been laid down by the Medical Officer of Health, Dr. Middleton

Martin, whereby the general practitioners were brought into relation with primary hospitals, and these primary hospitals were brought into relation with the secondary hospitals in the large towns.

The provision for the health of the people in hospital or otherwise was demonstrably inadequate. It needed not to be swept away but to be expanded and adjusted. Hospitals were inadequate because the population had grown more rapidly than the hospitals had. Again, recent progress in medicine demanded increased institutional treatment, which the present hospital system was not elastic enough to supply. The need was not merely one of providing more hospitals, but of the careful grading and linkage of those in existence.

The disability from sickness was a great weight upon the nation. In 1916 sickness benefit in England for men alone amounted to nearly 3½ millions sterling, representing 6,800,000 weeks of sickness and consequent withdrawal from employment. The average annual loss due to sickness and disablement in the case of both sexes for the years 1914–16 worked out at 270,000 years, and in these estimates all light sickness (of shorter duration than four days) was excluded, as well as all maternity cases and cases in sanatoriums. If an adequate health organisation existed these figures should at least be halved. For the full development of such an organisation the cost would be too great at present, and at the moment all that was asked was that stock should be taken of the position, that a review should be made of what existed at present compared with what should exist, and that a scheme be devised in outline sufficient to reveal the plan according to which the health organisation of the future should be shaped. There was already a patchwork of medical services—tuberculosis, venereal, and so on—which had come each in response to an urgent demand, and not as the result of any ordered policy. The time had come to consider the whole position and decide upon the lines of development, and then set to work cautiously and tentatively to build up the fabric of a medical organisation to approximate national needs. The Consultative Council was quite prepared to find that such a fabric could only be set up bit by bit. Certain principles must be laid down, but they must be principles which should be capable of some immediate applications, and he hoped that one such application might be made in Bristol. The burden of the report was simply this: the provision of fabric and equipment, together with freedom of action for doctor and patient. The general practitioner of the future must be more closely identified with preventive medicine and have access to the necessary equipment.

The full report of Lord Dawson's speech will be found in the

*British Medical Journal* for July 17th, but we may here draw attention to Lord Dawson's remarks anent the tendency for certain infirmaries to be handed over to the municipalities : "A municipalised service was one step forward to the nationalisation of medicine, and that would be a disaster alike to the medical profession and the public."

The Lord Mayor of Bristol proposed the health of representatives from neighbouring towns and counties ; and the Lord Mayor of Cardiff, in responding, supported the proposals set forth by Lord Dawson, but expressed the hope that the scheme would not be worked by existing local authorities—a separate *ad hoc* body should be created in the various districts.

A meeting of the Bath and Bristol Branch of the British Medical Association, held in the Council Chamber of the University, followed immediately after the luncheon and was largely attended.

The President, Dr. Watson-Williams, called on Lord Dawson, who more fully explained the proposals of the Consultative Council. It endeavoured to make the preventive and curative sides of medicine meet in the general practitioner, who must be looked upon more and more as the guide in matters of health of the residents of his district. The director of the communal clinic of the primary health centre should be a man whose practice was in the district ; his post should be a part-time one, paid on a time basis. This was the alternative to a whole-time salaried service. Training would be required for such a post, but this simply meant an extension of postgraduate instruction. As methods of investigation and treatment of disease became ever more complex the more did the insufficiency of the general practitioner's equipment become apparent. Nothing astonished him more as a consultant than the excellent work which was done by men under poor conditions. One of the vital parts of the organisation now foreshadowed was the full participation of the general practitioner in the communal equipment available at the health centre. The ideal primary health centre would have a certain number of rooms available for the examination of patients ; there would be X-ray provision, particularly for screen examinations, also facilities for blood testing, and so on.

Dr. J. Middleton Martin (Medical Officer of Health for Gloucestershire) gave an account of the plan developed in that county. While it was more or less a simple matter to provide in the towns the special services now required, the matter was more complicated in country districts. In Bristol, for example, there were 13,000 persons to the square mile, while in Gloucestershire, not one of the most sparsely-populated counties, there were only 268. It was out of the question to provide special services adequately for a scattered population on the same lines as for a compact population. It would be disastrous to the medical profession if continually branches of work were taken away from the general practitioner; nor would it be good for the community, because the preventive side of public health required a knowledge of the circumstances of the local community and the homes of the people, and who possessed this knowledge to the same extent as the general practitioner? Therefore the aim in Gloucestershire had been to bring the general practitioner into relation with what Lord Dawson called primary health centres. The attempt was made to develop the present communal services in centres where the local practitioners could deal with the cases. It would be mainly an out-patient service, but a certain number of beds were also wanted. The stations would be of a very simple character, but capable of development. The direction of these centres was an important consideration. There had been complaint in the past that medical schemes were under lay direction. Medical matters were so technical that there must be medical direction of such services, and from a plan already in operation in Cheltenham he had conceived the idea of setting up a medical advisory committee, composed, in the case of Gloucestershire, of the general practitioners in charge of the out-stations and the consultants at the hospitals. All medical matters would be referred to that committee, which would be the governing body in medical matters. He did not suggest that all difficulties had been overcome, but sufficient advance had been made to prove the practicability of the idea. As for finance, the complete scheme was not too expensive to be carried out at the present time. The money had been found in Gloucestershire by pooling the sums available for the separate schemes, and in this way sufficient money was found without increasing the estimates.

These introductory speeches were followed by a very interesting and fruitful discussion, to which Drs. M. Milligan (Bath), Bristowe (Wrington), Norgate (Bristol), Gordon (Exeter), Myles (Bristol), Newman Neild (Bristol), Luckham (Salisbury), Maclean (Cardiff), Flemming (Bradford-on-Avon), took part.

The discussion was briefly summed up by the President, who conveyed the thanks of those present to Lord Dawson for his address. Lord Dawson replied to various points raised in the course of the discussion, and emphasised particularly that the report of the Council did not aim at a State Service, he regarded their proposals as the sole alternative to a State Service. He stated that the Ministry of Health did not desire a bureaucratic control, but aimed at a health service of which the Ministry would be the inspirer, controller, and co-ordinator—that was all.

For a fuller report of this and the previous meeting reference should be made to the *British Medical Journal* for July 17th, 1920, upon which we have drawn largely for our notes.

---

**University of Bristol.**—Students of the University have recently passed the following examinations :—

University of Bristol. M.B., Ch.B.—*First Examination* : A. F. Alford, K. F. Alford, G. W. R. Bishop, O. J. P. Bollon, H. W. Brassington, D. E. Crellin, C. H. Durnford, F. S. Dymond, S. J. H. Griffiths, C. I. Ham, C. F. Hayhurst, H. F. Hollingshead, J. A. Hooker, C. T. Hyatt, C. F. R. Killick, A. H. Lowther, R. E. Lucas, A. C. Lysaght, E. May, W. S. Ormiston, Y. de la Pasture, M. P. Posthuma, D. C. Prowse, L. G. Robertson, H. Roger, H. J. Satchwell, R. E. Satchwell, J. F. Southward, G. M. Terrell, M. M. Te Water, W. Turner, T. W. Ware, T. M. White, K. M. Wilmore.

*Biology only* : S. P. Taylor, C. F. Wilson.

*Final Examination, Part I.* : W. H. Royal ; *Part II.* : T. H. A. Pinniger.

D.P.H.—*Part I.* : F. V. Cant, A. D. Fraser. *Part II.* : C. H. Hart, S. H. Kingston, A. D. Symonds.

B.D.S.—*First Examination* : E. K. Tratman.

*Zoology* (*completing examination*) : H. A. Jones.

*Third Examination* : G. N. Season.

Diploma in Dental Surgery.—*First Examination* : V. E. Allen, H. N. V. Barnes, A. S. C. Boulter, A. H. Butcher, R. G. Butterworth, L. H. Challenger, H. P. Evans, G. Holt, H. C. Hopes, K. A. Hunt, A. R. Hutchings, F. C. G. Lewis, J. C. W. Lewis, E. H. Longney, R. March, C. G. Payne, H. W. Pidgeon, M. Ritblat, J. R. Ritblat, G. G. Royal, C. E. Smith, W. H. Simpkiss, K. M. Watson, F. C. Winter.

*Third Examination* : W. Bayly, L. C. Bodey, E. E. Lewis, J. C. L. Phillips. *Final Examination* : E. J. Tucker.

M.R.C.S., L.R.C.P. Lond.—H. V. Jackson.

D.P.H. Oxon.—*Part I.* : M. S. Baker, C. Kingston.

L.S.A.—*Forensic Medicine* : J. L. Delicati.

